# Machine Learning Methods for Classifying Multiple Sclerosis and Alzheimer’s Disease Using Genomic Data

**DOI:** 10.3390/ijms26052085

**Published:** 2025-02-27

**Authors:** Magdalena Arnal Segura, Giorgio Bini, Anastasia Krithara, Georgios Paliouras, Gian Gaetano Tartaglia

**Affiliations:** 1 Centre for Human Technologies, Istituto Italiano di Tecnologia, Via Enrico Melen, 83, 16152 Genova, Italygiorgio.bini@iit.it (G.B.); 2 Department of Biology ‘Charles Darwin’, Sapienza University of Rome, P.le A. Moro 5, 00185 Rome, Italy; 3 Department of Physics, University of Genova, Via Dodecaneso 33, 16146 Genova, Italy; 4 Institute of Informatics and Telecommunications, National Center for Scientific Research “Demokritos”, 15341 Athens, Greece; akrithara@iit.demokritos.gr (A.K.); paliourg@iit.demokritos.gr (G.P.)

**Keywords:** machine learning, deep learning, multiple sclerosis, Alzheimer’s disease, logistic regression, extremely randomized trees, gradient-boosted decision trees, polygenic risk score

## Abstract

Complex diseases pose challenges in prediction due to their multifactorial and polygenic nature. This study employed machine learning (ML) to analyze genomic data from the UK Biobank, aiming to predict the genomic predisposition to complex diseases like multiple sclerosis (MS) and Alzheimer’s disease (AD). We tested logistic regression (LR), ensemble tree methods, and deep learning models for this purpose. LR displayed remarkable stability across various subsets of data, outshining deep learning approaches, which showed greater variability in performance. Additionally, ML methods demonstrated an ability to maintain optimal performance despite correlated genomic features due to linkage disequilibrium. When comparing the performance of polygenic risk score (PRS) with ML methods, PRS consistently performed at an average level. By employing explainability tools in the ML models of MS, we found that the results confirmed the polygenicity of this disease. The highest-prioritized genomic variants in MS were identified as expression or splicing quantitative trait loci located in non-coding regions within or near genes associated with the immune response, with a prevalence of human leukocyte antigen (HLA) gene annotations. Our findings shed light on both the potential and the challenges of employing ML to capture complex genomic patterns, paving the way for improved predictive models.

## 1. Introduction

In the context of genetics, complex diseases or conditions arise from the combined effects of multiple genomic variants and genes, are influenced significantly by both the physical and the social environment, and display non-Mendelian inheritance patterns. As a result, finding genomic factors that contribute to predisposition or protection against these diseases often requires large cohorts of individuals with comprehensive genomic information to obtain statistically significant results.

In recent decades, population genomics has made advances in the genomic characterization of complex diseases, mainly due to improvement in genomic technologies, an increase in the available genomic data, and the emergence of genome-wide association studies (GWAS) [1]. While the utility of GWAS is undeniable, several limitations make it challenging to identify the exact causal variant. The first is the presence of linkage disequilibrium (LD). Several studies used fine-mapping tools to address issues derived from LD and find the causal single nucleotide variants (SNVs) in GWAS signals [2]. However, the genomic variants associated with the disease selected by these tools are not always consistent across methods [1].

Another limitation of GWAS is that associations between SNVs and the phenotype are commonly tested using linear regression models for continuous phenotypes, or logistic regression models for binary phenotypes. Therefore, while GWAS effectively uncovers the main effects of genomic variants within LD blocks concerning a particular condition, it is less suited for detecting interactions between genomic variants influencing disease risk.

In population genomics, the most popular statistical approach used to quantify an individual’s genomic risk of developing a trait or disease is the polygenic risk score (PRS), which uses summary statistics obtained from GWAS to fit the equations. PRS has been extensively used in many studies, demonstrating its ability to extract disease risk propensity scores from diverse cohorts and conditions [3]. However, a limitation still exists, as PRS is not designed to detect epistatic events associated with a condition.

Alternatively, machine learning (ML) and deep learning (DL) are subfields of artificial intelligence (AI) that focus on the development of algorithms and models that enable computers to learn from and make predictions based on data. DL is a subset of ML that focuses on neural networks having multiple layers. The emergence of big data has facilitated the application of these methods, contributing to their increasing popularity in a wide range of fields, including population genomics. ML methods calculate the importance of input features during training and can detect interactions and complex patterns in the data. The use of Explainable AI (XAI) methods in ML aims to provide information on the most relevant features as considered by the models for making predictions [4,5]. In this context, the most informative genomic features identified by ML methods can foster the discovery of complex genomic profiles associated with diseases.

In this work, ML and DL methods were used to classify individuals with one of two complex diseases, multiple sclerosis or Alzheimer’s disease, compared to non-affected controls sourced from the UK Biobank (UKB), using data from genotyping arrays.

Alzheimer’s disease (AD) is a progressive neurodegenerative disorder characterized by cognitive decline, memory loss, and changes in behavior, primarily affecting older individuals. The disease is associated with the accumulation of abnormal protein deposits, including beta-amyloid plaques and tau tangles, in the brain. ML classifiers have been previously employed to classify AD using genotyping data [6,7,8,9,10,11]. In order to reduce the dimensionality of the genomic data, some studies applied pre-filtering of SNVs based on *p*-values from GWAS or other univariate tests [7,8,9]. In addition, XAI tools applied to DL models in AD have been evaluated in several studies [12,13,14,15].

Multiple sclerosis (MS) is a chronic autoimmune disease of the central nervous system where the immune system mistakenly attacks the protective covering of nerve fibers (myelin), leading to communication disruptions between the brain and the rest of the body. This can result in a wide range of symptoms, including fatigue, difficulty walking, numbness or tingling, and problems with coordination and balance [16]. Several studies have aimed to identify individuals with MS and their associated genetic loci linked to the disease and its progression by applying ML methods [17,18]. Additionally, the detection of epistatic events among genomic variants associated with MS using ML methods has been studied before [19]. To our knowledge, this is the first study to evaluate the classification of individuals with MS in the UK Biobank using genomic data and ML methods.

Overall, the application of ML methods to identify individuals with complex diseases based on genomic data has gained popularity in recent years. However, a limitation is that the published works on this topic are relatively recent, and there are not yet many studies evaluating the robustness of these methods. Additionally, some of the studies referenced in the previous lines used relatively small sample sizes in the analysis (fewer than 1000 cases per group), making it challenging to draw generalizable conclusions.

The primary objective of our study is to assess the variability and robustness of ML techniques in predicting complex diseases using genomic data. Specifically, we evaluated models and investigated performance differences among ML methods and both diseases. We also compared the performance of ML methods with PRS and assessed the influence of feature selection techniques on the model performance. The secondary goal of this study is to apply XAI tools to the ML models to extract information about the prioritized features that contributed the most in the classification task, pointing to potential predisposing or protective genomic variants in the disease.

## 2. Results

### 2.1. Performance of the Models

**Figure 1:**
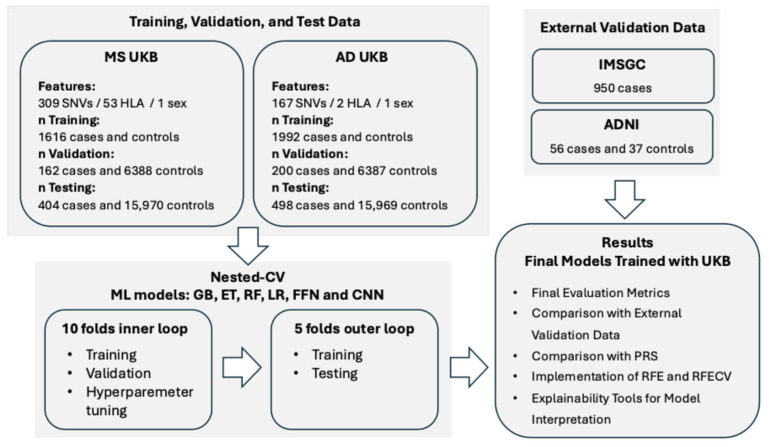
Design of the study. MS: Multiple Sclerosis; AD: Alzheimer’s Disease; UKB: UK Biobank; SNVs: Single Nucleotide Variants; HLA: Human Leukocyte Antigen; CV: Cross-Validation; GB: Gradient-Boosted Decision Trees; ET: Extremely Randomized Trees; RF: Random Forest; LR: Logistic Regression; FFN: Feedforward Neural Networks; CNN: Convolutional Neural Networks; PRS: Polygenic Risk Score; RFE: Recursive Feature Elimination; RFECV: Recursive Feature Elimination with Cross-Validation; IMSGC: International Multiple Sclerosis Genetics Consortium; ADNI: Alzheimer’s Disease Neuroimaging Initiative. More details can be found in the Section 4 and in the Supplementary Materials.

For this work, we used information from individuals in the UKB (Figure 1; Supplementary Table S1). The ML methods employed in this work were logistic regression (LR), three tree-based ensemble ML methods—Gradient-Boosted Decision Trees (GB) [20], Random Forest (RF) [21], and Extremely Randomized Trees (ET) [22]—and two DL methods: Feedforward Neural Networks (FFN) and Convolutional Neural Networks (CNN) [23] (Supplementary Table S7, Figures S3 and S4). The features used in the ML models were genomic variants associated with the disease reported in curated databases (Figure 1; Supplementary Tables S4–S6). To train the models, we balanced cases and controls with the different undersampling strategies described in the Supplementary Materials. We evaluated the final models using metrics that assess each class’s performance separately and are not influenced by class distribution in the dataset, including balanced accuracy, sensitivity, and specificity.

**Figure 2:**
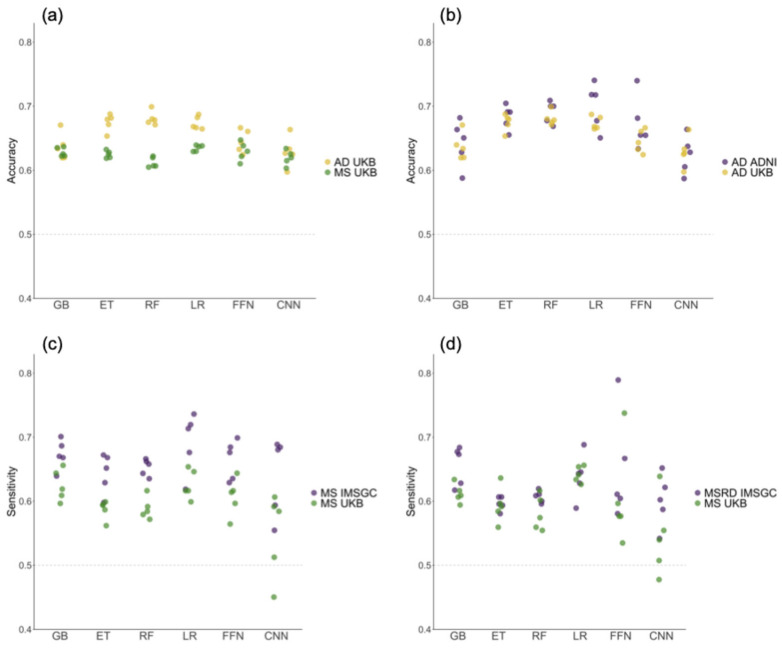
The values of evaluation metrics across the five folds of the outer loop in the nested cross-validation, obtained by training the models on the UK Biobank (UKB) cohort and testing them on different datasets. (**a**) The balanced accuracy obtained when testing on the UKB cohort. (**b**) The balanced accuracy values for the Alzheimer’s disease (AD) models tested on the UKB and the Alzheimer’s Disease Neuroimaging Initiative (ADNI) cohorts. (**c**) The sensitivity values for the multiple sclerosis (MS) models tested on the UKB and the International Multiple Sclerosis Genetics Consortium (IMSGC) MS cohorts. (**d**) The sensitivity values for the UKB and IMSGC MSRD cohorts. GB: Gradient-Boosted Decision Trees; ET: Extremely Randomized Trees; RF: Random Forest; LR: Logistic Regression; FFN: Feedforward Neural Networks; CNN: Convolutional Neural Networks.

In Figure 2a and in Table 1 and Table 2 we show the evaluation metrics for the MS and AD models we constructed. The mean performance scores for both diseases typically ranged between 0.6 and 0.7, with few exceptions. The FFN and CNN methods exhibited the least stable performance, as evidenced by the highest standard deviation (SD) across folds. In the case of AD (Figure 2a and Table 2), the GB method performed similarly to CNN and FFN, with low mean performances and high SD. Alternatively, GB demonstrated relatively good performance in MS (Figure 2a and Table 1).

Conversely, ET and RF were among the best methods in the context of AD and showed average performance for MS. These findings emphasize the variability in the performance of DL and tree-based methods when tested across various folds and diseases. For both diseases, the LR method exhibited low SD values while displaying consistent results across various evaluation metrics.

External validation datasets with cohorts from the International Multiple Sclerosis Genetics Consortium (IMSGC) and the Alzheimer’s Disease Neuroimaging Initiative (ADNI) were used to evaluate the models’ generalization performance for MS and AD, respectively (Figure 2b–d). Sensitivity and balanced accuracy did not decrease in external validation datasets compared to the reported results in the UKB test set, supporting the ability of all models to generalize across diverse datasets and populations. Furthermore, the use of external validation datasets allowed us to rule out overfitting concerns.

### 2.2. Comparison of Machine Learning Methods with Polygenic Risk Score

The performance of ML methods was compared to PRS. The same division of samples into folds was used for the PRS calculation and ML methods, ensuring the same individuals were compared across the five folds. The ML models employed in this work, which are classifiers, return the prediction scores assigned to each subject that range from 0 to 1. However, the PRS returns risk scores as continuous values without any fixed range. To convert a PRS into predicted classes, the standard practice is to set a cut-off using percentiles. In this work, we applied percentiles to the prediction scores obtained with ML methods and to the values of PRS to compare their performance. The relative risk (RR) and odds ratio (OR) were calculated by considering individuals having the highest values of PRS and ML scores (upper 99th percentile) as predicted positives (Table 3 and Table 4 for MS and AD, respectively). Values of RR and OR greater than one indicate a higher proportion of individuals with the disease in the upper 99th percentile compared to those in lower percentiles.

For the ML methods, results are similar to those obtained for the general evaluation metrics presented in Table 1 and Table 2, with LR performing relatively well across diseases. For MS, PRS was among the top three methods after LR and FFN, as shown in Table 3. In AD, PRS had average performance compared to the other ML methods, as shown in Table 4.

The ML methods were employed as classifiers in the previous section of this work, applying a cut-off prediction score of 0.5, where positives had values greater than or equal to 0.5, and negatives had values lower than 0.5. The RR and OR values considering the default cut-off used in the ML classification are provided in Table 3 and Table 4. FFN demonstrated the lowest SD across folds when considering the top 99th percentile of samples with the highest scores as predicted positives in MS (Table 3). The 99th percentile corresponded to prediction scores higher than or equal to 0.89 obtained with the FFN models. Conversely, FFN exhibited the highest SD when using the prediction score cut-off of 0.5. These results suggest that the FFN models applied to MS exhibited greater robustness in stratifying the positive class when a higher cut-off was used to split the classes.

**Figure 3:**
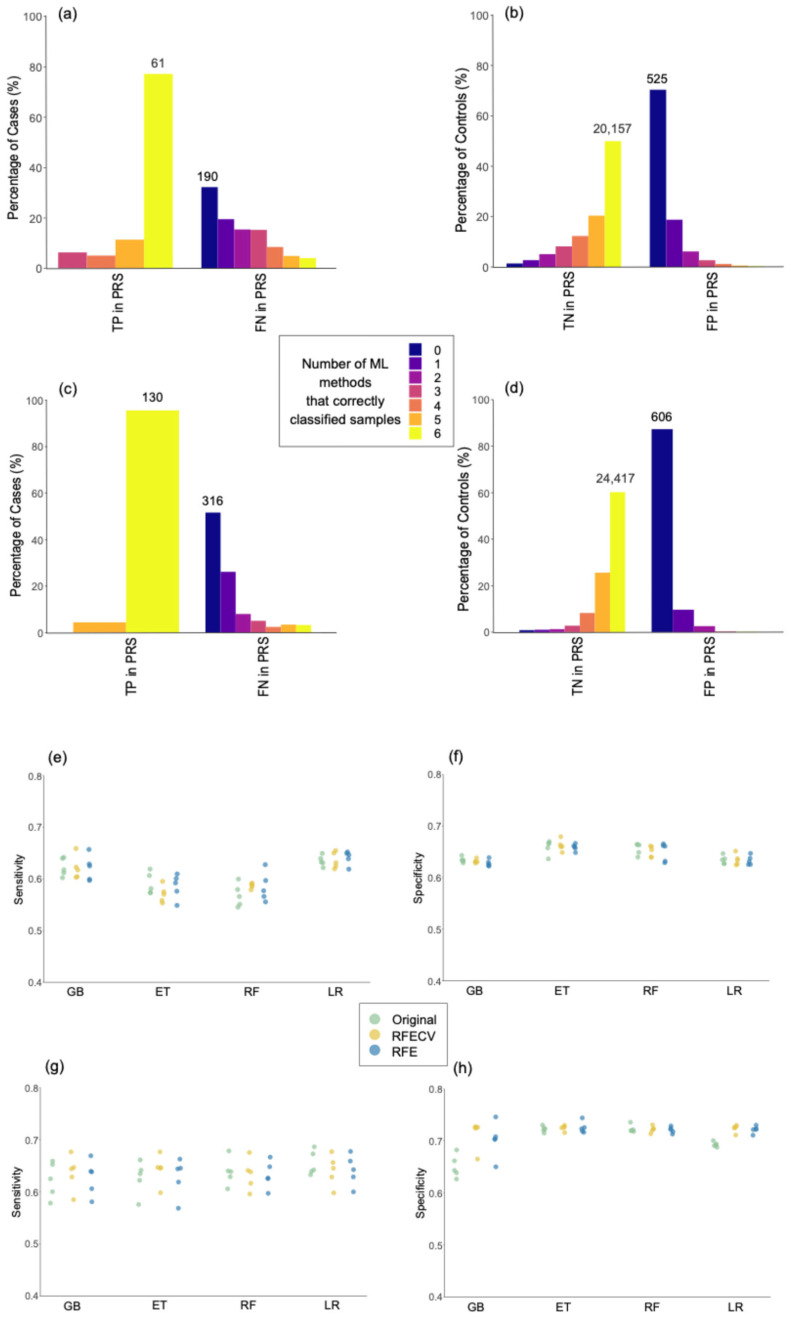
Assessing the robustness of predictive models. Percentage of controls correctly classified by 0 to 6 machine learning methods in comparison with samples correctly classified as true positives (TP) or true negatives (TN), and incorrectly classified as false negatives (FN) or false positives (FP) by polygenic risk score (PRS) models. The total number of samples is indicated above the bars for the groups having the highest percentage in each comparison. Panels (**a**,**b**) show the classification of cases and controls in multiple sclerosis (MS) models, respectively. Panels (**c**,**d**) show the classification of cases and controls in Alzheimer’s disease (AD) models, respectively. (**e**–**h**) Sensitivity and specificity in the original models, and models after feature selection with Recursive Feature Elimination with Cross-Validation (RFECV) and Recursive Feature Elimination (RFE). Sensitivity and specificity in multiple sclerosis are represented in plots (**e**) and (**f**), respectively. Sensitivity and specificity in Alzheimer’s disease are represented in plots (**g**) and (**h**), respectively. GB: Gradient-Boosted Decision Trees; ET: Extremely Randomized Trees; RF: Random Forest; LR: Logistic Regression.

We investigated whether individuals predicted as positives above the 99th percentile or as negatives below the 50th percentile by the PRS models were consistently classified as positives and negatives across the six ML methods using the standard 0.5 cut-off (Figure 3a–d). In MS, 77% of cases correctly classified as positives by PRS were also classified as true positives (TP) by the six ML methods, as indicated by the yellow bar in Figure 3a. Additionally, 70% of the samples classified as false positives (FP) by PRS were also misclassified by the six ML methods, as indicated by the dark blue bar in Figure 3b. In AD, the percentage of TP according to PRS with full agreement across ML methods was 96%, as indicated in the yellow bar of Figure 3c, while the percentage of FP by PRS with full agreement across ML methods was 87%, as represented by the dark blue bar in Figure 3d.

Overall, the results presented in this section suggest that, when evaluated based on percentiles, the performance of PRS is similar to that of the ML models. Additionally, the results in Figure 3 indicate that PRS and ML models demonstrate consistent classification outcomes, with similarities in the classification of specific individuals. The strengths and weaknesses of ML methods and PRS are further elaborated in the Section 3.

### 2.3. Implementation of Feature Selection Techniques

In this study, we used curated databases of disease-related genomic variants to select the predictors for the ML models. However, these databases contain genomic variants from diverse studies conducted in various human populations, some of which may not be informative in the UKB cohort. Furthermore, certain genomic variants used as features in models are highly correlated due to LD, which could potentially have a negative impact on the performance of the models. To address this, we used feature selection techniques such as recursive feature elimination (RFE) and recursive feature elimination with cross-validation (RFECV), aiming to identify a subset of features having the potential to enhance model performance. These methods enable recursive pruning based on the model’s intrinsic metrics while accounting for potential feature interactions [24]. Due to the considerable variability observed in the performance across folds by the DL methods, which could potentially compromise the robustness of the comparisons, and the fact that, in DL, the direct application of feature selection techniques is less common [23], these tools were not evaluated in the DL methods.

As shown in Figure 3e–h, there were no significant differences in sensitivity or specificity when comparing models after applying RFECV and RFE with the original models. However, reducing the number of features in the models with RFE and RFECV decreased the number of correlated features due to LD. This trend is represented in Figure S5 (Supplementary Materials). Therefore, the results suggest that the presence of correlations among the genomic variants did not significantly impact the models’ performance.

In the case of MS, 74% to 88% of samples were classified into the same class using the features in the original models, RFE, and RFECV, as shown in Table 5. The value is particularly high for AD (see Table 6), where, except for the GB method, around 93% of samples were classified as the same class using different sets of features. These results indicate that the original models, RFE, and RFECV lead to the same predictions for the majority of individuals.

With few exceptions, for MS (Table 5), the number of features selected by RFECV and RFE in each fold exceeded that for AD (Table 6). In fact, for AD, RFECV selected only one SNV in eight folds (highlighted in red in Table 6). As noted in the column labelled “n features 5 times”, 12 to 125 features were consistently selected across the five folds in MS (Table 5), whereas AD had only 1 to 5 features selected (Table 6).

For AD, the missense variant rs429358 in the Apolipoprotein E (*APOE*) gene was the one consistently chosen across all the folds using the various feature selection techniques and ML methods. It was also the only feature selected in the eight different folds following RFECV selection, as highlighted in red in Table 6. rs429358 is a SNV with the minor and major alleles being (C) and (T), respectively. The fact that RFECV proposed models with the variant rs429358 alone, without any noticeable impact on model performance, suggests that the majority of predictions were entirely influenced by this variant in the original models.

**Figure 4:**
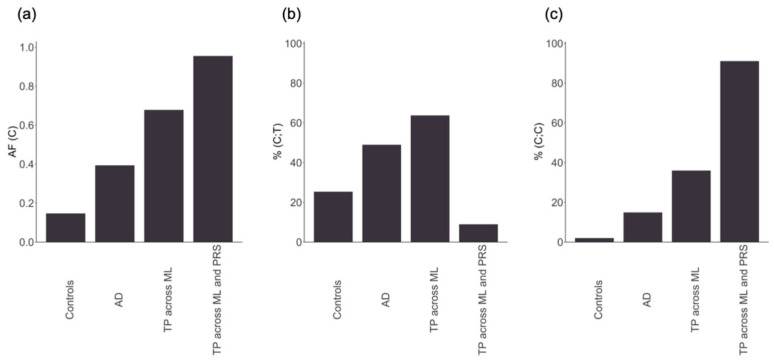
From left to right: (**a**) the allele frequency (AF) of the rs429358 (C) minor allele; (**b**) the percentage of individuals with the heterozygous form of the allele (C;T); (**c**) the percentage of individuals with the two copies of the minor allele (C;C). The x-axis represents controls, individuals with Alzheimer’s disease (AD), AD correctly classified by the six machine learning methods (TP across ML), and AD correctly classified by the six machine learning methods and the polygenic risk score (TP across ML and PRS).

To support this assumption, Figure 4 represents the allele frequency (AF) and the percentage of individuals with the rs429358 (C) allele, present in either the heterozygous or homozygous form, across different groups. In individuals with AD, the presence of rs429358 (C) or AF was more than double that in controls (Figure 4a). The AF progressively increased in individuals with AD classified as true positives across all ML methods and in those correctly classified by both ML and PRS. Among individuals with AD classified as true positives across all ML methods, 64% had (C;T) alleles (Figure 4b), while 36% had (C;C) alleles (Figure 4c). With the exception of three individuals with AD, all of them had at least one copy of the rs429358 (C) allele. In contrast, for individuals with AD consistently classified as AD across both ML and PRS, all had at least one rs429358 (C) allele, 91% had (C;C) alleles (Figure 4c), while 9% had (C;T) alleles (Figure 4b). Therefore, individuals with the highest risk of developing the disease according to PRS and ML methods appear to be those with (C;C) alleles, followed by individuals with (C;T) alleles, in an additive risk pattern. These results corroborate that the models constructed for AD predominantly relied on a single SNV, rs429358, and that the high consistency observed in the classification of individuals across different methods for this disease is primarily attributed to this variant.

For MS, the human leukocyte antigen (HLA) variant HLA-A*02:01 was the only genomic variant consistently selected across the different folds and methods. However, we discarded the possibility that the MS models relied solely on this variant for making the predictions, based on the fact that the number of features used in the MS models after feature selection ranged from 20 to 341 (Table 5), which supports the presence of polygenicity in MS, as previously reported in other studies [25].

### 2.4. Prioritized Genomic Variants in Multiple Sclerosis

To proceed with the use of explainability tools and to extract the importance of the features assigned by the models, we focused only on MS. AD was excluded from these analyses because, as demonstrated in the previous section, the classification for this disease relied heavily on a single SNV.

**Figure 5:**
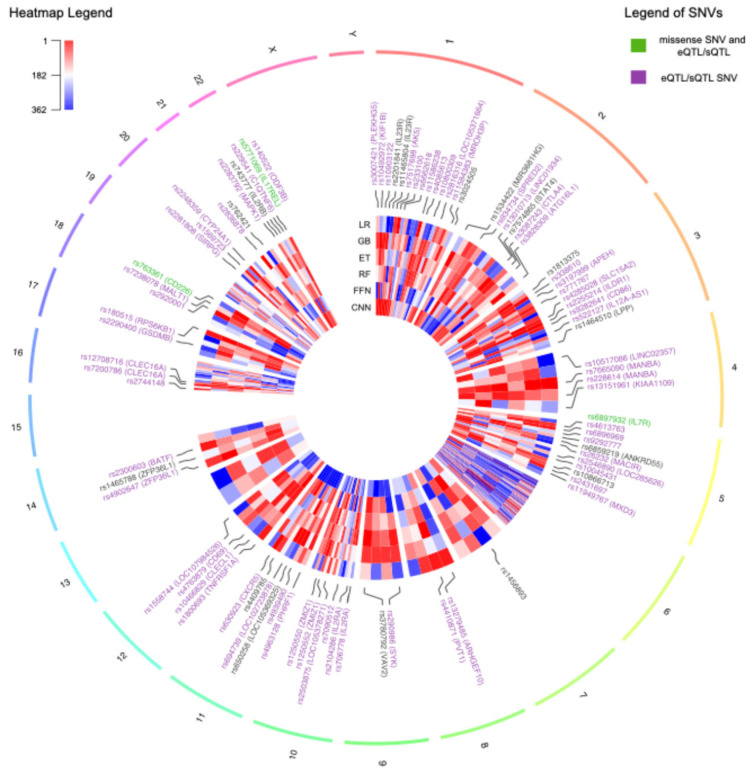
Circos plot representing all the genomic features used in the multiple sclerosis models distributed across the genome. The heatmap indicates the ranks of the features as assigned by each machine learning method, with values close to 1 in red indicating higher importance. The variants that were prioritized by at least one method are indicated with their names. The names of the single nucleotide variants (SNVs) are colored in purple if they are annotated with an expression quantitative trait loci (eQTL) or splicing quantitative trait loci (sQTL) in at least one tissue in the Genotype-Tissue Expression Portal (GTEx). The labels of missense SNVs with annotated QTLs are colored in green. The labels of chromosome 6 are excluded due to the high density of prioritized genomic variants in this chromosome. GB: Gradient-Boosted Decision Trees; ET: Extremely Randomized Trees; RF: Random Forest; LR: Logistic Regression; FFN: Feedforward Neural Networks; CNN: Convolutional Neural Networks.

**Figure 6:**
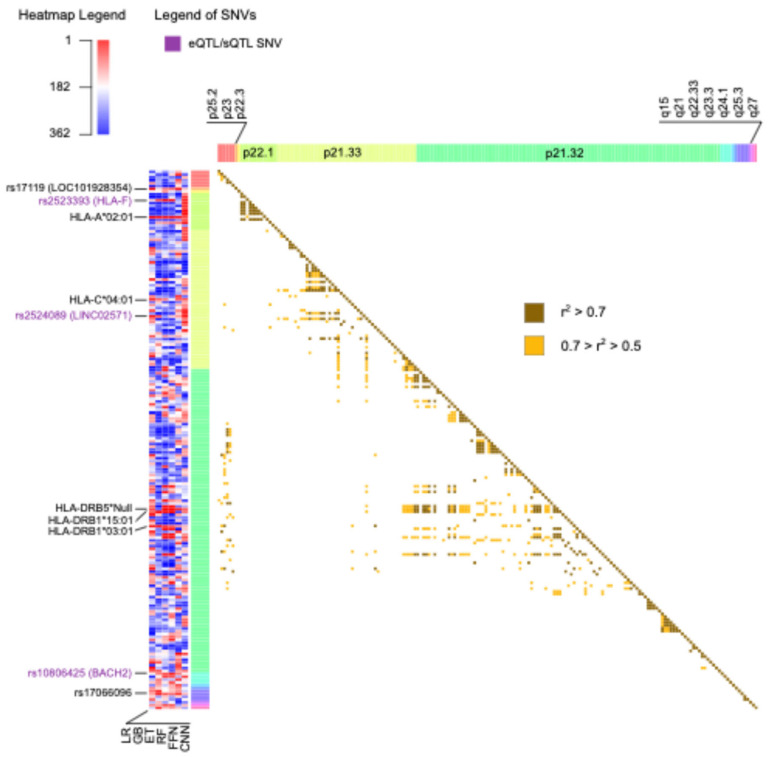
The heatmap on the left represents the ranks of all the features on chromosome 6 as assigned by each machine learning method, with values close to 1 in red indicating higher importance. The top 10 best-ranked genomic variants on this chromosome are labeled with their corresponding names. Labels in purple indicate the presence of an expression quantitative trait loci (eQTL) or splicing quantitative trait loci (sQTL) in at least one tissue in the Genotype-Tissue Expression Portal (GTEx). The heatmap on the right indicates the presence and strength of linkage disequilibrium between pairs of genomic variants. GB: Gradient-Boosted Decision Trees; ET: Extremely Randomized Trees; RF: Random Forest; LR: Logistic Regression; FFN: Feedforward Neural Networks; CNN: Convolutional Neural Networks.

For each ML model, the genomic variants were ranked using ordinal numbers, with values close to one representing higher importance (Supplementary Table S5). In total, there were 136 genomic variants that were among the top 10% with the best rank in at least one ML method, 50 of which were located on chromosome 6. From now on, these will be referred to as prioritized variants. The circos plot in Figure 5 includes a heatmap depicting the ranks and locations of all the genomic variants used as features in MS. The genomic variants that were prioritized by at least one method are annotated with their names, excluding those on chromosome 6. Due to the high density of prioritized genomic variants on chromosome 6, this chromosome is independently represented in Figure 6, along with the names of the 10 highest-ranked variants and the pairwise LD.

The heatmaps in Figure 5 and Figure 6 illustrate the substantial variability in the ranks assigned to genomic variants, often displaying diverse colors corresponding to the ranks obtained using the different ML methods. Notably, all missense variants used as features in the MS models were annotated as likely benign by AlphaMissense [26]. This finding aligns with the polygenic nature of MS, wherein the cumulative effect of numerous small to medium genetic effects across the genome predisposes individuals to, or protects them from, the disease [25]. Alternatively, most of the SNVs were predicted to have an effect in expression (eQTL) or splicing (sQTL), as annotated using GTEx and highlighted with purple labels in Figure 5 and Figure 6.

Three prioritized variants were missense SNVs, highlighted with green labels in Figure 5: rs6897932, located in the *IL7R* gene on chromosome 5; rs763361, located in the *CD226* gene on chromosome 18; and rs5771069, located in the *IL17REL* gene on chromosome 22. The *IL7R* gene encodes the interleukin-7 receptor, which is involved in the development and function of T cells. *CD226*, on the other hand, encodes a glycoprotein also known as DNAX accessory molecule-1 (*DNAM-1*), which plays a role in the regulation of T cell activation and the immune response. Additionally, genomic variants in *IL7R* and *CD226* have been associated with other autoimmune diseases, such as type 1 diabetes and rheumatoid arthritis [27]. It is worth noting that there was an absence of prioritized missense variants on chromosome 6.

The top 10 ranked genomic features on chromosome 6 were determined by summing the ranks obtained across the six ML methods and are labelled in Figure 6. There was not only one location on chromosome 6 associated with MS; instead, the top 10 risk loci were widely distributed across cytobands. Among the top genomic features on chromosome 6, there were five HLA types: HLA-A*02:01, HLA-C*04:01, HLA-DRB5*Null, HLA-DRB1*15:01, and HLA-DRB1*03:01. Additionally, the SNV rs2523393, located in *HLA-F*, was identified as both eQTL and sQTL for this gene. Furthermore, the SNV rs2524089, an intron variant in *LINC02571*, was recognized as an eQTL and sQTL for the genes *HLA-B*, *HLA-C*, and *HLA-E*. In this regard, the prevalence of HLA gene annotations among the top genomic features on chromosome 6 highlights their significance in the context of MS.

The top 10 best-ranked genomic features across all chromosomes are listed in Table 7. When considering all chromosomes, the highest-ranked genomic variant was HLA-A*02:01 on chromosome 6. In the UKB cohort, HLA-A*02:01 was more common in controls compared to individuals with MS, with a Fisher test *p*-value of 2.43 × 10^−19^. This observation aligns with its reported protective effect against MS in the literature [28,29]. HLA-A*02:01 was also recurrently selected across all folds and methods with the RFE and RFECV techniques in the previous section, emphasizing the relevance of this variant for predicting MS outcomes in the UKB cohort. The *HLA-A* gene belongs to the major histocompatibility complex (MHC) class I. It is worth noting that the most significant genetic factor associated with MS, as reported in the literature, is HLA-DRB1*15:01 [30], which is a predisposing HLA variant belonging to the MHC class II. In the UKB cohort, HLA-DRB1*15:01 exhibited the most significant differences in allele frequency between individuals having MS and controls, with a Fisher test *p*-value of 2.77 × 10^−101^. It was more prevalent in MS than in controls, consistent with its predisposing role. However, when considering rankings across all chromosomes, this variant was ranked 23rd.

The SNVs rs7665090 and rs2248359 listed in Table 7 are located downstream and upstream of the genes *MANBA* and *CYP24A1*, respectively. Specifically, rs7665090 serves as both an sQTL and eQTL for *MANBA*, which is an exoglycosidase found in the lysosome and is involved in immune system pathways [31]. Alternatively, rs2248359 functions as an eQTL for *CYP24A1*, which encodes a protein responsible for the catabolism of the active form of vitamin D [32].

The SNV rs180515 is situated in the 3′ UTR of the *RPS6KB1* gene and is annotated as both an eQTL and sQTL for this gene. *RPS6KB1* is actively involved in immune response pathways, particularly in the IL-4 signaling pathway, which has been associated with the progression of MS [33].

The variants rs1800693, rs2283792, and rs7200786 are located within the intronic regions of the genes *TNFRSF1A*, *MAPK1*, and *CLEC16A*, respectively. The SNV rs1800693 functions as both an eQTL and sQTL for *TNFRSF1A*. This gene encodes a member of the TNF receptor superfamily of proteins and plays a role in regulating the immune system and initiating inflammatory reactions [34]. The SNV rs2283792 serves as an eQTL for *MAPK1*, which is linked to MS due to its involvement in the MAPK pathways [35]. The SNV rs7200786 functions as both an eQTL and sQTL for *CLEC16A*, which is a direct regulator of the MHC class II pathway in antigen-presenting cells [36].

Overall, most of the highest-prioritized variants were identified as eQTLs or sQTLs located in non-coding regions within or near genes associated with the immune response and MS. Other SNVs were prioritized by the models but were not annotated as missense variants, eQTLs, or sQTLs affecting relevant genes. This could be partially attributed to the presence of LD, which results in highly correlated genotypes for variants located close together. While this correlation among features confers similar predictive power in the models, it does not necessarily imply that each individual variant is relevant to MS.

## 3. Discussion

In this work, we investigated different aspects concerning the application of ML methods for predicting MS and AD based on genomic features. It is important to acknowledge that assessing genomic predisposition to complex diseases, which do not adhere to classic Mendelian inheritance patterns, presents challenges. Concerning this, some ML methods have the ability to identify complex relationships in the data that traditional statistical methods may overlook.

The performance of ML models was evaluated and compared across folds, methods, and diseases. The comparison of our results with other studies using ML to classify AD and controls is challenging due to substantial variability in performance across studies, likely caused by differences in ML methods, training approaches, evaluation metrics, genomic data, and populations [6,7,8,10,11]. For MS, a previously published study that applied ML methods to genomic data for classifying cases and controls reported a performance similar to that observed in our work [17].

Lower variability is often desirable in ML analyses, as it suggests more stable and reliable performance. Notably, DL methods exhibited the highest variability across folds. This could be attributed to the relatively modest sample size employed in this study, which poses challenges for generalization, especially when leveraging the deeper connections inherent in DL models. Related to this, several studies have suggested that less sophisticated ML methods tend to outperform DL methods when dealing with small sample sizes [37]. Supporting this notion is the fact that LR exhibited stable performance across folds and diseases and was consistently positioned among the top-performing methods. LR is known for its relatively simple algorithm and ease of implementation. Nevertheless, additive regression models such as LR, by default, are designed to detect main effects, preventing them from capturing interactions between input variables. In this respect, this limitation did not seem to negatively impact the results of LR in the current work.

Another relevant aspect we investigated in our work is how ML methods compare to PRS in stratifying individuals by disease risk. Once the conclusion has been drawn that PRS is comparable to ML methods, the question arises of whether to select one method over the other. ML offers several advantages over PRS. For example, PRS is limited to capturing only linear relationships with the disease, as its core algorithm is a linear additive model. In contrast, ML methods, with the exception of LR, can capture complex interactions and nonlinearities. This ability could be especially valuable for detecting synergisms between genomic variants that may go unnoticed using PRS. Additionally, in the case of ML, the interpretability of the model is more flexible compared to PRS. This is because PRS provides a risk score based on a set of genomic variants with their associated weights obtained from GWAS summary statistics, and these weights remain unmodified. However, ML methods have the capacity to learn from the data and refine the weights assigned to genomic variants during training, following different architectures depending on the method used.

PRS offers some advantages over ML. One of them is that there is no need to access large datasets with individualized genomic data to build the models. This is because PRS requires GWAS summary statistics, which are typically anonymized to protect the confidentiality of the study participants, facilitating their public availability [38]. Additionally, in PRS there are no limits to the number of genetic variants that can be included in the models. Therefore, some of the problems associated with the dimensionality of the data in ML are resolved. Finally, PRS fell within the average performance when compared across methods and diseases. In contrast, the performance of ML methods was more variable, especially among tree-based ML and DL methods.

It is worth noting that, apart from the methods used in this study, there is an extensive list of other ML methods that could be employed as classifiers, but it was not feasible to test all of them in this work. The uncertainty regarding the best ML method to use for solving a particular problem creates the necessity of testing several methods in the same study, adding complexity to the process of analyzing the data. In this context, previous studies have explored the application of ensemble methods to leverage the complementary strengths of ML methods and PRS, potentially improving model robustness [39,40]. This represents a promising avenue for future research.

In this study, we employed two recursive feature elimination tools, RFE and RFECV, to test whether a subset of predictors could enhance the models’ performance. After applying RFE and RFECV, the number of features in the models decreased, which also reduced the number of correlated features. Interestingly, there were no significant changes in the models’ performance after feature selection. This suggests that the LD among genomic variants did not majorly impact the performance of the original models. Robustness to LD has previously been reported for RF [41] but was unexpected for LR, which can be negatively affected by multicollinearity [42].

All the models developed in this study for AD predominantly relied on a single SNV, namely rs429358, for making predictions. After the application of RFECV techniques, some models relied exclusively on this genomic variant for classification, with no significant impact on performance. Located on chromosome 19 in the *APOE* gene, the allele (C) of rs429358 is one of the most extensively reported factors associated with AD risk and dementia, exhibiting an additive risk pattern [43]. The recurrent selection of rs429358 across methods and folds, over other variants in strong LD such as rs4420638 (r^2^ = 0.708) and rs769449 (r^2^ = 0.743), underscores the capability of RFE and RFECV to discern the most significant genomic variants linked to the disease, despite feature correlations. The strong association between rs429358 (C) in *APOE* and the disease might overshadow other, weaker genetic risk factors in the AD models. To account for additional genomic variants conferring small risk effects for AD, it is common practice to exclude the *APOE* region from GWAS and PRS calculations, treating the *APOE* locus as an independent factor or covariate [44]. The same approach could be applied to ML, ensuring enough representation of the three major ApoE isoforms.

For MS, we explored the ranking of genomic features assigned by different ML models. The diversity in the ranking of features across methods and folds highlighted the complexity of polygenic diseases. Additionally, the presence of LD likely contributed to the instability of the importance assigned to the genomic predictors [45]. In light of this, extracting general rules, such as a unique prioritization of features that applies to all methods, becomes challenging. Generally, the top genomic variants ranked by the ML models were located in non-coding regions near genes involved in the immune response or associated with MS. Moreover, most of these variants were annotated in GTEx as eQTLs or sQTLs for these genes in at least one tissue. There was an enrichment of HLA gene annotations among the prioritized genomic variants on chromosome 6. HLA-DRB1*15:01, belonging to the MHC class II genes, is the strongest genetic determinant of MS as defined in the literature [30]. However, in our work, the most consistently prioritized genomic variant in MS was HLA-A*02:01, which belongs to the MHC class I. Remarkably, there is evidence of the independent association of HLA-A*02:01 and HLA-DRB1*15:01 with MS [46], with the former considered protective and the latter predisposing to the disease, aligning with the results of our work.

The well-known region strongly associated with MS, in the vicinity of HLA-DRB1*15:01 on chromosome 6, did not seem to overshadow the relevance of other variants located on different chromosomes. Consistent with this observation is the fact that, when exploring the genomic variants within the top 10 by sum of ranks, only HLA-A*02:01 belonged to chromosome 6.

It is important to note that modifying any of the parameters in this study, such as employing different ML methods, selecting different genomic features, or applying ML methods to other diseases, could potentially alter some of the conclusions drawn in the current study. Indeed, one of the weaknesses of the ML methods studied in this work is their variability when certain conditions in the analysis are changed. Despite this limitation, ML methods have proven to be powerful and efficient in various everyday applications. In light of these trends, with the reduction in genome sequencing costs and improvements in sequencing technologies, the volume of genomic data is expected to continue increasing in the coming years. Simultaneously, the rise in computational capacities and advancements in existing ML methods are likely to increase the robustness of these methods and foster exciting discoveries in the field of population genomics.

## 4. Materials and Methods

### 4.1. Inclusion and Exclusion Criteria

The UKB was the main source of genomic data for this work. Additionally, the study conducted by IMSGC [47], available in dbGAP under the accession ID “phs000139.v1.p1”, was used as an external validation dataset for MS. The ADNI database (adni.loni.usc.edu) was used as an external validation dataset for AD. Details on the inclusion and exclusion criteria used to select cases and controls in each dataset are provided in the Supplementary Materials. Further details on the number of individuals from the UKB, IMSGC, and ADNI selected for this work, across diseases, after applying the selection criteria, are also provided in Figure 1, Figure S1 and Tables S1–S3 (Supplementary Materials).

### 4.2. Pre-Processing of Genomic Data

Of all the genomic variants present in the UK Biobank Axiom Array, the Affymetrix GeneChip^®^ Human Mapping 500K for the IMSGC dataset, and the whole-genome sequencing (WGS) data for ADNI, only those that were reported in ClinVar [48] with at least one level of review status, or those reported in the curated DisGeNet dataset [49] associated with the diseases under study, were used as predictors in the ML models. A binary feature indicating sex was also included in the models. When an HLA gene was associated with the disease, the imputed HLA types for this gene obtained from the UKB (UKB Field ID 22182) were included as predictors. The number of predictors used in each disease is shown in Figure 1 and Table S4 (Supplementary Materials), and the list of dbSNP IDs used as predictors for each disease is provided in Tables S5 and S6 (Supplementary Materials).

Genetic variants were encoded as 0, 1, 2, and 3, corresponding to a missing value, the absence of the variant, the presence of the variant in one allele, and the presence of the variant in two alleles, respectively, assuming an additive model. Genomic variants with the same values in all samples (monomorphic predictors) were excluded from the analysis. PLINK [50] was used for quality control. SNVs with a Hardy–Weinberg equilibrium *p*-value lower than 1 × 10^−8^, minor allele frequency lower than 0.05, missingness per marker higher than 0.2, and samples with missingness per individual higher than 0.2, were excluded. Additionally, PLINK was employed to compute the LD statistics between genomic variants.

The pipeline for imputing missing values involved several steps, and only the genomic variants that were already present in the UK Biobank Axiom Array but had missing genotypes in some samples (less than 20% of samples for each SNV after QC filters) were imputed. Haplotype phasing was performed using SHAPEIT4 [51], while IMPUTE5 [52] was employed for genomic imputation. The reference files for genomic imputation were obtained from the 1000 Genomes Phase3 [53]. Imputed genotypes with less than 80% probability were considered as missing, and imputed genomic variants with a quality score lower than 0.90 were excluded from further analysis (see the Supplementary Materials for more details).

### 4.3. Machine Learning Methods

Nested cross-validation (nested CV) was applied with 10 folds in the inner loop and 5 folds in the outer loop to select the optimum hyperparameter configuration and obtain an estimate of the model’s generalization performance. For the hyperparameter selection, the grid search approach was employed, and the 10 evaluation scores obtained for each hyperparameter configuration in the inner loop were used to select the optimal configuration. From the top 10 hyperparameter configurations having the highest values of balanced accuracy mean, the configuration having the highest value of sensitivity minus the SD of sensitivity across the 10 folds was selected. For each fold in the outer loop, the selected hyperparameter configuration in the inner loop was applied in the outer loop using 80% of balanced samples for training and 20% for testing. The strategy of nested CV used in this study is represented in Figure S2 (Supplementary Materials). Further details including the architecture of the DL models and the tested hyperparameters are provided in Figures S3 and S4, Tables S7 and S8 (Supplementary Materials). For feature selection, RFE and RFECV were used, employing the balanced accuracy as scoring function.

### 4.4. Explainability Methods Applied to Machine Learning Models

For GB, ET, and RF, feature importance was determined using built-in importance metrics. For LR, feature importance was based on feature coefficients. In DL methods, specifically FFN and CNN, importance metrics were derived using layer-integrated gradients (LIG) [54]. Features were ranked by importance in all models, and the top 10% of features from the model with the highest balanced accuracy for each ML method were identified as prioritized genomic variants associated with the disease. Further details can be found in the Supplementary Materials.

### 4.5. Polygenic Risk Score

PRS was computed using PRSice-2 software [55], with PLINK files from the UKB as the target data. GWAS summary statistics for MS [56] and AD [57] were sourced from the NHGRI-EBI GWAS Catalog [58] and used as base data. Covariates, including sex and the first 10 principal components from the UKB, were included in the PRS models. PRS was calculated five times for each disease, including in the regression model the same samples that were used in the outer loop of the nested CV for training the final ML models. The aim was to compare the performance of PRS with ML using the same samples for fitting and evaluation in each fold. To convert PRS into binary categories, a threshold was established to distinguish the individuals having a high risk of the disease. Individuals with a PRS above the 99th percentile were classified as high risk (positives). Similarly, the 99th percentile was applied to the probability scores obtained from ML models to classify high-risk individuals and to compare the results with the PRS models. The RR and OR were used to evaluate the models, with the formulas provided below:

$$RR=\frac{P^{99th}/(P^{99th}+N^{99th})}{P^{\prime }/(P^{\prime }+N^{\prime })}$$

$$OR=\frac{P^{99th}/N^{99th}}{P^{\prime }/N^{\prime }}$$

where P^99th^ and N^99th^ represent, respectively, the number of positives (individuals with the disease) and negatives (controls) present in the top 99th percentile with the highest PRS, or probability scores in the case of ML methods. P′ and N′ represent, respectively, the number of positives and negatives present in the samples that were not in the top 99th percentile. The number of SNVs selected for each fold after C+T and the corresponding PRS statistics are provided in Tables S9 and S10 (Supplementary Materials) for MS and AD, respectively.

## Figures and Tables

**Table 1:** The mean and standard deviation of evaluation metrics across the five folds in the outer loop of the nested cross-validation corresponding to the multiple sclerosis models. The accuracy reported in the table corresponds to balanced accuracy. SD: Standard Deviation; GB: Gradient-Boosted Decision Trees; ET: Extremely Randomized Trees; RF: Random Forest; LR: Logistic Regression; FFN: Feedforward Neural Networks; CNN: Convolutional Neural Networks.

	Accuracy Mean	Accuracy SD	Specificity Mean	Specificity SD	Sensitivity Mean	Sensitivity SD
GB	0.628	0.007	0.635	0.005	0.622	0.017
ET	0.625	0.006	0.660	0.014	0.590	0.022
RF	0.612	0.008	0.657	0.011	0.567	0.022
LR	0.635	0.005	0.635	0.008	0.634	0.010
FFN	0.629	0.014	0.599	0.059	0.660	0.075
CNN	0.619	0.011	0.652	0.058	0.587	0.067

**Table 2:** Following the same structure as Table 1, this represents the evaluation metrics for the Alzheimer’s disease models.

	Accuracy Mean	Accuracy SD	Specificity Mean	Specificity SD	Sensitivity Mean	Sensitivity SD
GB	0.637	0.021	0.651	0.022	0.623	0.034
ET	0.675	0.013	0.723	0.006	0.627	0.031
RF	0.681	0.011	0.723	0.007	0.639	0.026
LR	0.674	0.010	0.693	0.005	0.655	0.024
FFN	0.645	0.018	0.693	0.068	0.598	0.072
CNN	0.629	0.024	0.665	0.043	0.594	0.034

**Table 3:** The mean and standard deviation (SD) of the relative risk (RR) and odds ratio (OR) across the samples used in the five folds in the multiple sclerosis models. The formulas used in the calculation of RR and OR are provided in the Section 4. In the case of machine learning (ML) methods, the cut-off of probability, 0.5, which is the default in these methods, was also considered to calculate the RR and OR and is represented in additional columns. GB: Gradient-Boosted Decision Trees; ET: Extremely Randomized Trees; RF: Random Forest; LR: Logistic Regression; FFN: Feedforward Neural Networks; CNN: Convolutional Neural Networks; PRS: Polygenic Risk Score.

	RR Mean 99th Percentile	RR SD 99th Percentile	OR Mean 99th Percentile	OR SD 99th Percentile	RR Mean ML Classification	RR SD ML Classification	OR Mean ML Classification	OR SD ML Classification
GB	3.598	1.575	3.901	1.822	2.790	0.162	2.868	0.170
ET	3.886	1.653	4.247	2.004	2.719	0.119	2.795	0.125
RF	3.341	1.687	3.610	1.915	2.456	0.159	2.517	0.168
LR	5.509	1.390	6.232	1.782	2.935	0.119	3.021	0.126
FFN	4.107	0.630	4.455	0.752	2.908	0.426	2.988	0.444
CNN	2.827	1.060	2.984	1.219	2.641	0.269	2.712	0.284
PRS	4.002	0.729	4.333	0.864	---	---	---	---

**Table 4:** Following the same structure as Table 3, this represents the evaluation metrics for the Alzheimer’s disease models.

	RR Mean 99th Percentile	RR SD 99th Percentile	OR Mean 99th Percentile	OR SD 99th Percentile	RR Mean ML Classification	RR SD ML Classification	OR Mean ML Classification	OR SD ML Classification
GB	4.313	0.823	4.795	1.037	3.013	0.564	3.128	0.609
ET	6.643	1.315	8.031	1.914	4.183	0.426	4.408	0.462
RF	6.837	0.719	8.273	1.044	4.391	0.415	4.636	0.453
LR	6.882	0.651	8.336	0.969	4.094	0.368	4.300	0.398
FFN	5.906	1.696	7.011	2.528	3.350	0.611	3.502	0.673
CNN	4.788	1.798	5.473	2.487	2.862	0.608	2.971	0.661
PRS	5.683	1.098	6.644	1.523	---	---	---	---

**Table 5:** The table shows, for each machine learning model in multiple sclerosis, from left to right, the percentage of samples that were classified with the same class in the original models and models after feature selection, the distinct methods used for feature selection, the number of features in folds from one to five after feature selection, and the number of features selected from one to five times across different folds. GB: Gradient-Boosted Decision Trees; ET: Extremely Randomized Trees; RF: Random Forest; LR: Logistic Regression; RFE: Recursive Feature Elimination; RFECV: Recursive Feature Elimination with Cross-Validation.

	% Samples with the Same Prediction in Original, RFE and RFECV	Method	Features Fold1	Features Fold2	Features Fold3	Features Fold4	Features Fold5	n Features 1 Time	n Features 2 Times	n Features 3 Times	n Features 4 Times	n Features 5 Times
GB	78.31	RFE	150	250	250	250	200	54	33	40	65	120
		RFECV	314	138	252	321	341	19	29	56	124	125
ET	75.76	RFE	200	200	150	50	200	42	40	52	88	34
		RFECV	86	316	274	175	277	33	29	87	109	68
RF	74.10	RFE	100	250	100	100	200	62	66	38	28	66
		RFECV	225	103	216	79	251	30	49	85	44	63
LR	88.35	RFE	200	200	20	250	50	89	127	76	21	13
		RFECV	205	140	25	231	39	102	128	50	18	12

**Table 6:** Following the same structure as Table 5, this represents the values for the Alzheimer’s disease (AD) models. The values in red correspond to the single nucleotide variant rs429358, selected across all folds and methods for AD.

	% Samples with the Same Prediction in Original, RFE and RFECV	Method	Features Fold1	Features Fold2	Features Fold3	Features Fold4	Features Fold5	n Features 1 Time	n Features 2 Times	n Features 3 Times	n Features 4 Times	n Features 5 Times
GB	74.68	RFE	5	150	5	5	5	140	9	1	1	1
		RFECV	1	29	1	1	1	28	0	0	0	1
ET	93.02	RFE	150	5	100	100	5	34	35	75	4	3
		RFECV	32	1	1	2	1	30	1	0	0	1
RF	94.38	RFE	150	5	100	100	100	32	16	22	75	5
		RFECV	139	3	130	124	99	7	11	29	91	3
LR	92.23	RFE	20	5	20	50	20	29	21	10	1	2
		RFECV	7	1	7	51	3	44	4	4	0	1

**Table 7:** The top 10 best-ranked genomic features across all methods with the corresponding ranks assigned by each machine learning method. The values of the prioritized ranks are highlighted in red. dbSNP: Single Nucleotide Polymorphism Database; GB: Gradient-Boosted Decision Trees; ET: Extremely Randomized Trees; RF: Random Forest; LR: Logistic Regression; FFN: Feedforward Neural Networks; CNN: Convolutional Neural Networks.

dbSNP ID	Gene	Chromosome	LR Rank	GB Rank	ET Rank	RF Rank	FFN Rank	CNN Rank	Sum of Ranks
HLA-A*02:01	*HLA-A*	chr6	1	9	8	8	12	46	1
rs2255214	*ILDR1*	chr3	10	17	15	13	1	56	2
rs7665090	*MANBA*	chr4	16	5	112	18	32	54	3
rs180515	*RPS6KB1*	chr17	27	12	29	58	29	92	4
rs1800693	*TNFRSF1A*	chr12	35	93	36	29	3	68	5
rs2248359	*CYP24A1*	chr20	18	6	126	64	4	49	6
rs11586238		chr1	4	82	35	54	48	127	7
rs2283792	*MAPK1*	chr22	78	48	113	99	23	9	8
rs7200786	*CLEC16A*	chr16	17	27	17	30	42	239	9
rs4285028	*SLC15A2*	chr3	22	13	144	34	6	156	10

## Data Availability

Additional data will be provided upon request to the corresponding author, subject to controlled access policies of the source databases. All the scripts employed in this work are available at https://github.com/machalen/ML_complex_diseases (accessed on 25 February 2025).

## Supplementary Materials

The following supporting information can be downloaded at https://www.mdpi.com/article/10.3390/ijms26052085/s1. References [59,60,61,62,63,64,65,66,67] are cited in the supplementary materials.
